# Unpacking the dynamics of generative AI use in our daily lives: towards an integrative trust calibration framework

**DOI:** 10.1007/s00146-026-03279-0

**Published:** 2026-08-11

**Authors:** Theo Araujo

**Affiliations:** Amsterdam School of Communication Research (ASCoR), University of Amsterdam, Amsterdam, The Netherlands

**Keywords:** Generative AI, Trust, Conversational agents, Reliance, Technology use

## Abstract

The growing use of conversational generative AI agents by an increasingly diverse set of individuals gives rise to the following question: How do individuals calibrate their trust in a dynamic, seemingly general-purpose technology that learns from and adapts to their behavior and may take an increasingly central role in their online interactions? In this article, we propose the first steps towards an integrative framework for trust calibration in genAI agents focusing on individuals using these agents in their daily lives. We address the notion of calibrating trust in technology, reviewing five challenges to our understanding of trust calibration in such context, namely (1) the complex nature of individual private use, (2) the complexity in assessing *actual* trustworthiness, (3) the fuzzy notion of the *trustee*, (4) the complex ontological status of these agents, and (5) the *over-time* nature of trust calibration. We conclude with a set of propositions for trust calibration in genAI agents and considerations for future research.

## Introduction

The release of generative AI (genAI) in the form of conversational agents has reshaped the communication environment and the daily routines of an increasing share of individuals. GenAI agents such as ChatGPT, Claude, Gemini or DeepSeek (among others) represent a qualitative shift in the capabilities of earlier conversational agents: they can arguably hold longer and more natural conversations with users (Bansal et al. 2024) and generate credibly looking responses, even if those responses are not always accurate, appropriate or normatively acceptable (Bender et al. 2021; Weidinger et al. 2022). This shift in capabilities is reflected in how individuals use these agents in their daily lives, often going beyond the text or image generation activities for which the technology may have originally been designed (Skjuve et al. 2024; Moravec et al. 2024; Chatterji et al. 2025). GenAI agents have thus potentially impacted how an increasingly larger group of individuals learn, work, seek information or advice, organize and perform daily activities, and even form relationships—with other humans, or with the agents themselves.

These developments bring forth a crucial question: how do individuals calibrate their trust in a dynamic, seemingly general-purpose technology that learns from and adapts to their behavior, may take an increasingly central role in their online interactions, and is deployed by digital platforms with goals and interests of their own? Understanding trust calibration is a key priority of research about technology use (Allan et al. 2021; Wischnewski et al. 2023; Mehrotra et al. 2024), especially if we hope to avoid the threat of *technology disuse*—whereby individuals distrust all genAI agent interactions outright and miss their benefits—and of *technology misuse—*with individuals subjected to unprecedented levels of manipulation due to naïve, over-trust.

In this article, we propose the first steps towards an integrative framework for trust calibration in genAI agents focusing on individuals using these agents in their daily lives or, in other words, when they act as “non-expert users” of this technology (as defined by, e.g., Papagni et al. 2023). To develop such a framework, we outline a series of theoretical and empirical challenges that must be overcome if we are to understand how trust calibration takes place. In the sections that follow, we address the notion of calibrating trust in technology, how it may need to be revisited given challenges posed both by characteristics of these agents and by their varying contexts of use, and propose an integrative framework ultimately aimed at empowering individuals to calibrate their trust in this technology as it becomes increasingly pervasive in their lives.

## Calibrating trust in generative AI

Trust may be conceptualized as the willingness to depend on another party in the face of at least some level of uncertainty (Rousseau et al. 1998; Gambetta 2000; Mcknight et al. 2011; Bodó 2020), often characterized as a judgment (Levi and Stoker 2000; Smith 2025). Specifically for technology, trust has been further defined as the evaluation “that an agent will help achieve an individual’s goals in a situation characterized by uncertainty and vulnerability” (Lee and See 2004, p. 51). It is judgment of a *trustor* towards a *trustee*. In these conceptualizations, we find the first set of questions when it comes to calibrating trust in genAI agents. On the one hand, even if one does not consider genAI agents as being a general-purpose technology (as argued, for example, by Eloundou et al. 2024), individuals do use these agents for a diverse set of motivations and purposes (Skjuve et al. 2024; Moravec et al. 2024). Not all types of use may therefore be equally characterized by uncertainty or vulnerability and, as such, when can we speak of trust, and are there differences in how it is formed and conceptualized across use contexts? On the other hand, the notion of a *trustee*, in itself, may be fuzzy: is the trustee the agent, as a stand-alone entity (e.g., ChatGPT or Google Gemini), is it the technology (i.e., Generative AI as an umbrella term, or even LLMs as the underlying technology), or the companies (e.g., OpenAI, Google) that make these agents available to wide range of individual users? Or a combination of them?

Furthermore, it has been argued that one can only *rely* on technology, but not *trust* it (Deley and Dubois 2020). This follows from the notion that one can only *trust* another human (Baier 1986), as the trustor must be able to assign intent to—and feel betrayed by—the trustee. However, as Wischnewski et al. (2023) have argued, given our tendency to anthropomorphize automated systems, we may move from the notion of *reliance* towards the notion of *trust*, whereas Lee and See (2004), among others (see Saßmannshausen et al. 2023), position trust as guiding reliance. The notion of *trust*, furthermore, may be even more relevant for genAI agents, as individuals need only minimal cues to anthropomorphize conversational agents (Araujo 2018), and tend to respond to technology as an independent source of information (Sundar and Nass 2000). The ontological nature of these digital interlocutors, however, may be seen differently by individuals (Guzman 2019), who, in the past decades, have developed more nuanced scripts to interact with technology in their lives (Gambino et al. 2020). This brings us to the second set questions for trust calibration in this context: is the genAI agent seen as a tool—i.e., an inanimate technological artifact—or is it an interaction partner? In this sense, can we trust these agents as independent entities, or merely rely on them?

The final concept that requires unpacking is the notion of *trust calibration* itself. Calibrated trust has been broadly defined as the attribution of *appropriate levels* of trust to a system, in line with its capabilities and limitations (Parasuraman and Riley 1997; Lee and See 2004). One can speak of calibrated trust when the *perceived* trustworthiness of a system—i.e., as perceived by the trustor—is in line with the *actual* trustworthiness of such a system—i.e., the trustee (Wischnewski et al. 2023). The notion of calibrated trust in AI has also been referred to as the “Goldilocks zone of credibility”, with individuals neither under- nor overestimating AI capabilities (Allan et al. 2021). More generally, calibration is an essential aspect to help prevent both the misuse and the disuse of technology (e.g., Lee and See 2004; Hoff and Bashir 2015; Glikson and Woolley 2020). Behind the notion of trust calibration often stands the assumption of an individual acting an *operator* of one *tool* aiming to achieve one specific (often work-oriented) *goal* (Lee and See 2004; Hoff and Bashir 2015; Glikson and Woolley 2020; Lockey et al. 2021). This says little about calibrating trust on a technology that can be used for multiple purposes, some of which, as we argue below, may yield the assessment of *actual* trustworthiness almost impossible. Moreover, the way in which AI interactions are shaped—via explanations communicated by the agent, its earlier performance, human-like cues or data collection processes—create a unique set of *trust-challenges*, i.e., trust-related risks and vulnerabilities (Lockey et al. 2021) yet to be examined empirically in an integrative manner (Lockey and Gillespie 2024). These challenges are further reinforced by the notion that trust calibration is an over-time process (Lee and See 2004; de Visser et al. 2020; Schuetz et al. 2025), with potentially different mechanisms than those for *enhancing* trust (Zhang et al. 2020). The multipurpose nature of this technology, and its unique set of trust-challenges give rise to the question: to what extent can we speak of *actual* trustworthiness for genAI agents—as a pre-requisite for trust calibration—and how does trust calibration take shape as a dynamic, interactive process, across different contexts of use?

The questions outlined above highlight the need to refine and extend our knowledge about trust calibration in genAI agents, specifically focusing on the experience and usage by individuals in their daily lives. To do so, a set of theoretical and empirical challenges that, we argue, call for an integrative framework to understand trust calibration in genAI agents for individual, private use. We work out each of these challenges in the section that follows, propose the first steps towards an integrative framework, and conclude with an outlook for further research.

## Conceptualising use—and trust—in generative AI agents: the challenges

Understanding trust calibration in genAI agents for individual, private use requires, first, specifying the notion of *use* itself. A long tradition of technology use research—including established models such as the Technology Acceptance Model (TAM; Davis 1985) or the Unified Theory of Acceptance of Technology (UTAUT; Venkatesh et al. 2003)—tends to operationalize *use* as the general activity of engaging with the technology—either by *adopting* it and/or by *continuing to engage* with it in an over-time manner. Wald et al. (2025) have extended this conceptualization beyond *use-frequency* (i.e., engagement with the technology in general) and included, among other concepts, *use purpose*, i.e., for what purpose or specific tasks the technology is used.

Specifically for genAI agents, studies highlighted both utilitarian and hedonic purposes of use (Skjuve et al. 2023) and developed taxonomies of a wide range of *use purposes* (e.g., Skjuve et al. 2024; Moravec et al. 2024; Flaßhoff et al. 2025), including, information and obtaining new knowledge, productivity for work or school, novelty and exploration, entertainment, fun and amusement, learning, and development, social interaction, companionship and support. This wide range of use purposes is also seen in analyses by the companies offering these services themselves, with, for example, OpenAI-related research indicating that a majority of user messages with ChatGPT are about information-seeking, practical guidance, self-expression, help with technical issues, and multimodal content (Chatterji et al. 2025). In sum, individuals interact with genAI agents in their daily lives for a complex and varied set of use purposes, thus raising a set of challenges when one considers how individuals may calibrate their trust in this technology.

### Challenge 1: the complex nature of individual, private use

The complex and varied nature of individual use of genAI agents implies that understanding trust formation and development in a genAI agent may require a nuanced approach, for two reasons. First, because the strength and the type of vulnerability may differ if, for example, a genAI agent is used for emotional support compared to information seeking—and even within information seeking, the type of information also plays a role. This becomes even more pressing as these technologies, when used for information seeking, may take the role of mediators of truth (Shin 2025). In some cases, one may even wonder the extent to which trust is a relevant concept, for example, when the use purpose is entertainment. In short, as proposed by Dahlin (2025, p. 6094), “different things will matter in different situations that include AI systems, and therefore we need to be open for a range of variations of what is needed to trust AI”. This further reinforces the need to conceptualizing risk when analyzing trust relations (Schuetz et al. 2025) which, in the case of genAI agents, may be uneven—or even non-existent—depending on the use purpose.

Second, individuals also systematically differ in how they use genAI agents, in multiple ways. For example, individual characteristics often associated with the digital divide literature (such as socioeconomic status) also seem relevant to understand differences in use-frequency and use purpose of genAI agents (e.g., Kacperski et al. 2024; Zhang et al. 2026), with trust itself also potentially having a role (as in Zhang et al. 2026). In addition, individuals vary widely in their levels of AI literacy (Wang et al. 2024), which has been shown to influence attitudes (such as satisfaction) towards genAI agents, depending on the use purpose (Lee and Park 2023). The importance of understanding these differences is further highlighted by decades of research on trust in technology (Glikson and Woolley 2020; Yang and Wibowo 2022; Saßmannshausen et al. 2023; Bach et al. 2024; Mehrotra et al. 2024; Schuetz et al. 2025): This research consistently points to the importance of individual differences—including personal characteristics, previous experiences, ability and attitudes—as well as the context of use for the understanding how trust is formed and developed.

Ultimately, the diverse nature of genAI use challenges a central notion followed by much of the research on trust in technology: That the individual is an *operator* of one *tool* aiming to achieve one specific (often work-oriented) *goal* (Lee and See 2004; Hoff and Bashir 2015; Glikson and Woolley 2020; Lockey et al. 2021). This assumption is challenged by how individuals use genAI agents in their private lives, especially given the diversity of use purposes, and their varying levels of risk. Moreover, the way in which AI interactions are shaped—the explanations communicated by the agent, its earlier performance, human-like qualities or data collection processes—create a unique set of *trust-challenges*, i.e., trust-related risks and vulnerabilities (Lockey et al. 2021) that are yet to be examined empirically in an integrative manner (Lockey and Gillespie 2024). The challenge then becomes understanding how trust calibration takes place across use purposes, by individuals with varying levels of motivation, ability or knowledge to critically assess their outputs and recommendations.

### Challenge 2: what is *actual* trustworthiness?

The complex nature of individual, private use also brings forward a new challenge: assessing *actual* trustworthiness of these systems, including which trustworthiness dimensions may be relevant, and how. Trust calibration has usually been conceptualized and studied by understanding how the usage of a system changes trust levels over time, compared to the actual reliability of a system (Wischnewski et al. 2023; Mehrotra et al. 2024). In other words, calibrated trust is usually seen as when “the perceived trustworthiness of a system matches the actual trustworthiness of a system” (Wischnewski et al. 2023, p. 2). Actual trustworthiness, in the case of AI systems, encompasses both technical aspects about its performance *and* matters related to its ethical acceptability and societal impact (Zanotti 2025).

And, it is precisely in the assessment of actual trustworthiness of genAI agents for private use that lies one of the largest challenges in studying trust calibration. As argued above, individuals use this technology for a variety of use purposes. One can argue that these agents therefore suffer from a “curse of flexibility”, with issues in requirements and complexity in the software making it challenging to understand what the software should or should not do (Leveson 2011)—as discussed by Lindström et al. (2025) in the context of the way in which the models behind genAI agents are trained. This curse is potentially at play when considering how to assess the *actual* trustworthiness of these systems: the multipurpose nature of the technology may imply that determining their *actual* trustworthiness would require such an assessment, at the limit, to be done *per* use context, with specific dimensions of trustworthiness—such as safety, accuracy, fairness, auditability, among others^1^—taking different shapes or levels of importance depending on the purpose of use.

Moreover, the answers provided by these agents are also highly dependent on the way in which individuals formulate their requests (e.g., Jang and Lukasiewicz 2023), with their consistency varying even by language or topic (Zhou and Zhang 2024). This is important, as individuals may vary widely in how they interact with genAI agents—for example, in the prompting strategies they adopt when seeking information (Xue et al. 2026)—which subsequently shapes the types of answers they receive and, arguably, how trust in these agents may be formed or developed. In addition, these answers may also change, arguably, across time—with the introduction of new models or data. This points to a second related issue in assessing these systems actual trustworthiness: would it be possible to discuss actual trustworthiness per use purpose, or would one need to assess the actual trustworthiness for *each* answer a genAI agent provides, contextually, for *each* user?

### Challenge 3: the fuzzy notion of a trustee

Another challenge with trust calibration in genAI agents is the question of who individuals consider the *trustee* to whom they may assign their trust. In the case of (gen)AI, one can speak of a network of actors who have agency—and may be attributed responsibility (Taddeo and Floridi 2018), arguably, functioning as the trustee. These actors may be articulated in terms of the company that provides the AI service (e.g., OpenAI, Google, among others) or the much wider network of organizations operating at global scale which provide the AI infrastructure (Crawford 2021; Mügge et al. 2026). They may also be articulated in terms of the individual roles formally involved with the process of creating an AI model or service, such as data scientists, software engineers, business owners, or data providers (see, e.g., Sullivan and Fosso Wamba 2022), not to mention the hidden work from platform workers who train and refine AI models (Gray and Suri 2019; Morgan et al. 2023).

This complexity is not necessarily new, as trust in technology research has previously focused on trust in the organization behind the technology, in the technology itself, or in the technological artifact (for an overview, see Schuetz et al. 2025). What may be particularly relevant in this context is, first, the even more prominent role of the technological artifact (i.e., the genAI agent) as a trustee, given individual tendencies towards source orientation (Sundar and Nass 2000)—i.e., perceiving a technological artifact as an independent source of information—as well as the complex ontological status of these agents, with some individuals seeing them as distinct entities—and as such examples of the medium becoming the communicator (Zhao 2006; Peter 2018)—or others as being part of the device or the technology more broadly (as seen, for example, with earlier versions of virtual assistants: Guzman 2019). A second element that may be particularly relevant in this context are the sources of the information (or answer) being provided by these agents in their answers (Jacques et al. 2026), which may become a new set of actors in the potential network of trustees. Finally, a third element that may play a role is the broader institutional infrastructure behind these systems, encompassing both in the form of the organization behind a specific technological artifact (e.g., OpenAI in the case of ChatGPT), or a more diffuse, broader network of organizations behind the technology. In this sense, individuals may not be calibrating their trust only towards an agent or its output, but also towards an organization or even towards broader institutional claims about safety, security, and trustworthiness of the technology,^2^ with an open question as to the extent to which different individuals may find these claims credible or even be aware of them. These claims include, for example, the “logic of AI safety securitization”, with organizations admitting that there are concerns with the technology, and making speech acts showing that there are actions (such as red teaming practices or research ongoing to address such challenges; Spelda and Stritecky 2025). In sum, this potentially complex network of trustees reinforces the need to carefully consider the referent when analyzing trust relations (as called for by, e.g., Wingert and Mayer 2024), which therefore becomes even more important in the case of the fine-grained, complex nature of use of genAI agents by individuals in their daily lives.

### Challenge 4: the complex ontological status of genAI agents

The even more prominent role of the technological artifact (i.e., the genAI agent) as a trustee, outlined above, opens a series of new challenges when investigating trust calibration, especially when considering in “who” or in “what” individuals may be placing their trust, and what type of trust. GenAI agents may present themselves as friendly assistants, or (socially) intelligent entities with a strong emphasis in anthropomorphic features, as, for example, found in a visual analysis of ChatGPT self-portrayals (van Es and Nguyen 2025), thus further blurring ontological boundaries between humans and machines by emulating more human-like qualities (as seen in earlier versions of virtual assistants, e.g., Guzman 2020). Even in media coverage, the technology itself is assigned an active role when it comes to responsibility and, in some cases, even an explicit—and autonomous—role in terms of accountability (e.g., Saurwein et al. 2025). For some, these agents may be conceptualized as *otherware*, i.e., a technology that is not “tool-like” but rather may be perceived as a social counterpart which is also not human (Hassenzahl et al. 2020; Saßmannshausen et al. 2023). Others also warn that individuals may develop synthetic relationships with these agents, in which “continuing associations between humans and AI tools that interact with one another wherein the AI tool(s) influence(s) humans’ thoughts, feelings and/or actions” (Starke et al. 2024, p. 1834).

These blurring ontological boundaries have a special importance for trust. Earlier conceptualizations of trust in technology (e.g., Madhavan and Wiegmann 2007; Mcknight et al. 2011) have positioned it as differing from trust in other humans. This stream of research has at times adapted the *ability*, *benevolence*, and *integrity* framework (Mayer et al. 1995) into dimensions that may be relevant for machines, such as *functionality*, *helpfulness*, and *reliability* (for an overview, see Schuetz et al. 2025). Others (e.g., Lankton et al. 2015) have argued for matching the level of human-likeness of a technology to the trusting beliefs that may matter in a given context, instead of adopting an either-or approach.

This combination of *human-like* and *machine-like* (or functionality) trust has been used to study AI as a general technology (Choung et al. 2023), whereas studies directly studying these agents have largely focused on *human-like* constructs (e.g., Huynh and Aichner 2025) or combined multiple dimensions (as in e.g., Choudhury and Shamszare 2024; Bouyzourn and Birch 2025). This partly follows from the notion that many trustee-related factors—such as transparency, reliability, or performance—may be attributed to both humans and machines (Hancock et al. 2023). The complex ontological nature of these agents, which may differ across contexts of use as well as across individuals, requires therefore a careful conceptualization of the type of trust that may be relevant for each type of use, and for each type of user.

### Challenge 5: the *over-time* nature of trust (calibration)

Finally, trust is dynamic and develops across time (Boyle and Bonacich 1970; Mayer et al. 1995; Hoff and Bashir 2015; Long and Sitkin 2024). Research on trust in technology further reinforces the over-time, dynamic nature of trust (e.g., Glikson and Woolley 2020; Schuetz et al. 2025), often differentiating between initial levels of trust and how it further evolves (Lee and See 2004; Papagni et al. 2023). Likewise, trust calibration in technology is part of this over-time process (Schuetz et al. 2025), as individuals correct their over- and under-trust across time and interactions, based on their experiences with the system (Mehrotra et al. 2024). This is also seen in longitudinal studies on genAI agent usage (e.g., for education: Polyportis 2024), which point to over-time use-and-trust dynamics.

The mechanisms for trust calibration in genAI agents, however, are far from straightforward, and at times may be different from the mechanisms for enhancing trust. A case in point is the provision of explanations by the technology (Wischnewski et al. 2023), arguably one of the key approaches to assist users in establishing the trustworthiness of such systems (Shin 2021a, b; Papagni et al. 2023). These explanations at times help calibrate trust (Ghai et al. 2020), or may lead to over-trust (Suresh et al. 2020). While explanations can be influential in individuals’ trust journeys (Holliday et al. 2016), their timing and context may be crucial in assisting users (re-)calibrate their expectations and trust in the system (Papagni et al. 2023). Likewise, individual-level differences, such as access to AI literacy information, may also have an undesired effect and at times lead to unwarranted trust (Lockey et al. 2021). These over-time dynamics are, therefore, a central challenge in understanding how individuals calibrate their trust in these agents.

## Towards an integrative framework of trust calibration

As individuals interact with genAI agents in their daily lives for an increasingly wider variety of use purposes, it becomes crucial to understand how they can be empowered to adequately calibrate their trust in these agents if we are to minimize the risks of technology misuse and disuse. The five challenges outlined above call for an integrative model for understanding trust calibration in genAI agents.

**Fig. 1:**
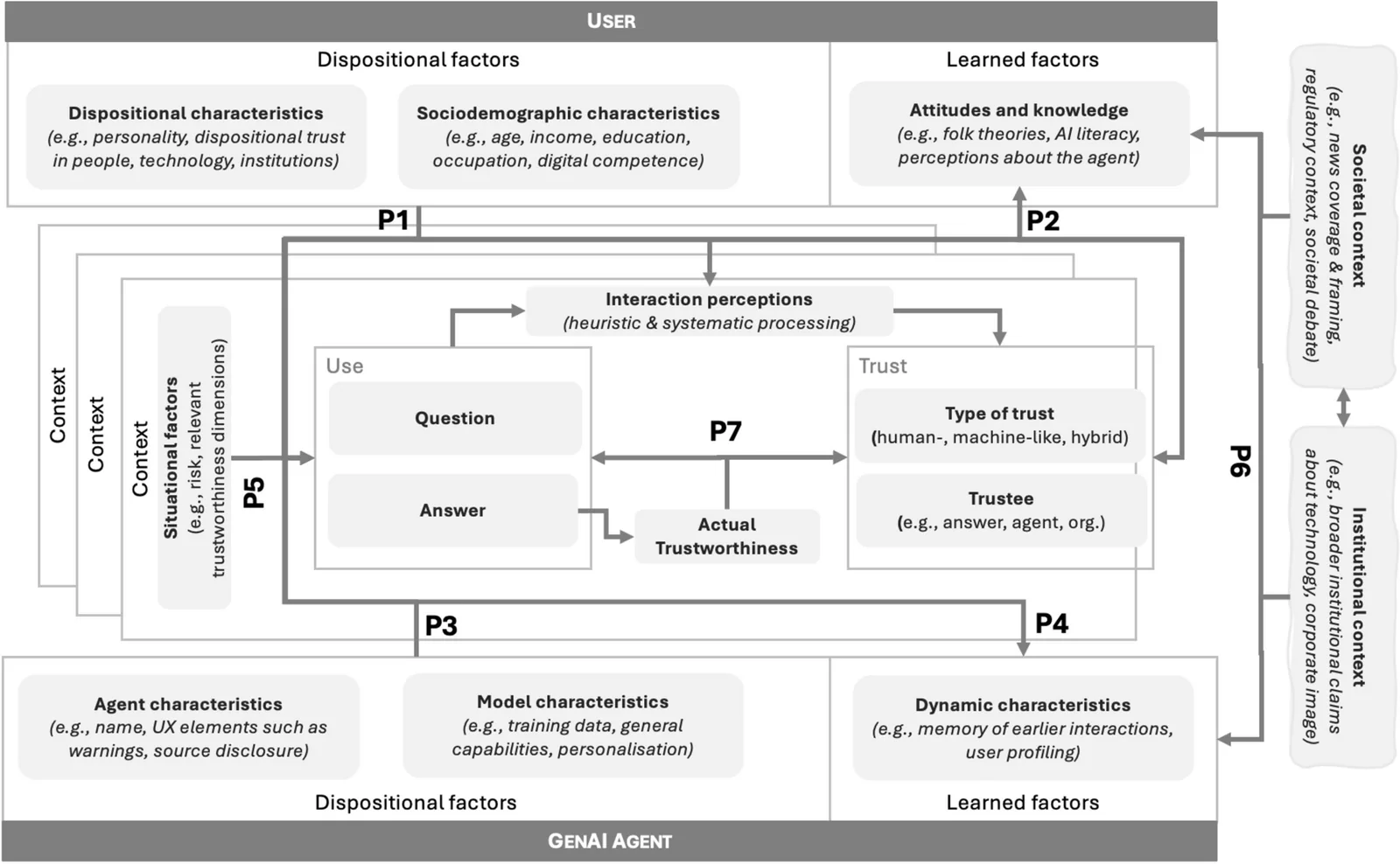
Towards an integrative framework for trust calibration in genAI agents

The first steps towards such an integrative framework, which we propose in Fig. 1, can build upon the body of knowledge on trust in technology (e.g., Lee and See 2004; Hoff and Bashir 2015; Schuetz et al. 2025) and trust in AI (e.g., Glikson and Woolley 2020; Lockey and Gillespie 2024; Dang and Li 2025). Specifically, it should account for the influence of factors related to the *individual*, of the *system* and of the *interaction*. Importantly, as acknowledged in the research streams reviewed above, the proposed model should consider trust calibration as a process that is both *ongoing* and *context-dependent*, towards a complex, multidimensional *trustee*. Core within this framework is the *trustor*, an individual who, as per dual-process models for understanding user interactions with (media) technology (e.g., Sundar 2020), may engage both in systematic processing of the information they receive, and in heuristic processing of cues in this information and in the system more broadly, which subsequently may influence their attitudes, behavior, and trust levels. Moreover, the elements of such an integrative framework encompass factors that may be categorized as dispositional (i.e., stable factors), situational (i.e., associated with the context of use) and learned (i.e., associated with the use of the specific system). With this, we adopt the framework proposed by Hoff and Bashir (2015), applying it not only to the user but also to the agent, given the ability from these agents to learn from earlier interactions and adjust their behavior to the user.

The proposed integrative framework visualized in Fig. 1 consists of seven propositions about trust-and-use dynamics of genAI agents by individuals and their influence on trust calibration dynamics. These propositions integrate individual, system, and interaction characteristics and consider the multipurpose nature of the technology and thus the notion that the individual (i.e., the trustor) may interact with the same agent across different contexts of use. We now turn to each proposition and, to make them concrete, we discuss relevant concepts that may be applied in two contrasting contexts, namely *information seeking*, in this example seeking factual, verifiable information, and *emotional support*, in this example interacting with the agent to reduce feelings of loneliness. Importantly, these propositions suggest factors that future research should account for when investigating trust calibration in genAI agents by individuals for private use yet are not an exhaustive list: They require validation and future research, as outlined in the discussion section.

### Individual dispositional factors

First, as proposed by trust in technology research (e.g., Hoff and Bashir 2015; Glikson and Woolley 2020) and other streams of scholarship, stable factors such as dispositional characteristics (e.g., personality traits, dispositional trust in technology, among others) and sociodemographics (as suggested by, e.g., digital divide scholarship; van Deursen et al. 2019; Kacperski et al. 2024; Wang et al. 2024) should be considered when investigating trust and trust calibration. These factors may influence the contexts in which individuals use these agents (i.e., use purpose): some individuals may be more prone to use them for information seeking, whereas others for emotional support, for example. The same factors may also shape how individuals interact with these agents (e.g., how they formulate their questions, how much and how often they engage in dialogue, etc.). They may also influence perceptions about the interaction and about the agents themselves. Finally, these factors may influence the *extent* to which individuals trust these agents, as well as the *way* in which individuals assign their trust, both in terms of which *trustees* become relevant for the individual (e.g., the agent, the organization, the answer, or the sources within the answer) and the *type* of trust that may be elicited (e.g., human-like, machine-like, or other configurations). This leads to the first proposition:

**P1.** Individual dispositional factors shape trust calibration via their influence on how individuals use, trust, and perceive interactions with these agents.

When applying this proposition to specific use contexts, it is important to note that a baseline set of dispositional factors (e.g., education levels, age, dispositional trust in technology, in people or in institutions, and digital competence) may influence how individuals interact with technology in general, yet potentially with different effects and directions depending on the context. Other factors, however, may become more relevant depending on the use context. For example, need for cognition (Cacioppo et al. 1996) or need for closure (Webster and Kruglanski 1994) may need to be taken into account for *information seeking*, whereas certain Big Five personality traits (McAdams and Pals 2006) such as openness, agreeableness, or neuroticism may influence how individuals will use genAI agents for *emotional support*. These generalized and context-specific factors may influence not only the extent to which individuals may use genAI agents for one of these purposes, but may subsequently influence how they use and how they assign trust to these agents.

### Individual learned factors

Individual dispositional factors are necessary, yet not sufficient to explain how individuals use and trust these agents across multiple interactions: One can expect that individual knowledge about and attitudes towards these agents will develop over time. This means that models for trust calibration should account for how individuals conceptualize and understand these agents—such as the role of algorithmic imaginaries and folk theories (Bucher 2019; DeVito 2021; Dogruel 2021)—as well as their knowledge, for example, their digital literacy and competence (Ferrari and Punie 2013; Wang et al. 2024; van Oosten et al. 2025). These learned factors are expected to influence use, trust in and perceptions about these agents, and to evolve as individuals continue interacting these agents. As such, we propose:

**P2.** Individual learned factors shape trust calibration via their influence on how individuals use, trust, and perceive interactions with these agents, with these factors subsequently being influenced by use.

These learned factors may be broadly applied across use contexts, as the same individual (i.e., the trustor) is learning about the agent across contexts of use, by, for example, developing their own folk theories about how these agents work, developing their digital competence based on earlier interactions (by, e.g., learning how to use better prompts to achieve their goals), or using the earlier performance by the agent as a baseline for expectations for future interactions (e.g., the role of prior experience; Sundar 2020). In certain contexts, however, specific factors may be more relevant or applied in different ways. Agent perceptions of anthropomorphism (Epley et al. 2007) may, for example, influence the extent to which individuals self-disclose to the agent (Papneja and Yadav 2025), and as such be relevant for *emotional support* as a context of use, and less so for *information seeking*.

### System dispositional factors

Understanding trust calibration also requires accounting for stable factors from the system. Agent characteristics such as its name or other characteristics in the user experience such as disclosures (e.g., “this agent can make mistakes”) or explanations (given their potential influence on user perceptions; Wang et al. 2026), or even its avatar (Ma et al. 2025) may influence how individuals use these agents, directly or via individual perceptions (e.g., heuristic processing of anthropomorphic/agency or credibility cues; Sundar 2008), therefore influencing trust (Sundar 2020). The same can be said for model characteristics, such as which type of training data was used or general model capabilities (e.g., displaying sources for the answer, searching online for new content etc.), which have a direct influence on the answers that the agent gives, thus influencing user perceptions about the agent and trust. As such, we propose:

**P3.** Agent dispositional factors shape trust calibration via their direct influence on how individuals perceive these agents and indirectly via the answers it provides during the interaction.

These factors are also expected to be applied across a wide range of use contexts, as they are related to baseline characteristics of these agents (e.g., the agent’s name, or a standard disclosure that it can make mistakes) or of their models (e.g., the capability for personalization or citing sources). However, they may have differential effects depending on the use context. For example, one could expect that citation of sources may have a larger role in how individuals assign trust to genAI agents for *information seeking*, given that displaying the original source of a content may serve as a heuristic that impacts credibility (Sundar 2008), which may be less relevant for *emotional support*. The general usage of human-like cues in language (e.g., referring to oneself as “I”, or usage of language cues that may trigger perceptions of anthropomorphism; Araujo 2018), however, may be more relevant for *emotional support*. Other factors, such as tendency for sycophancy, may affect both contexts (Sun and Wang 2026), though via different mechanisms.

### System learned factors

From the system side, the content of earlier interactions with the same individual may be used to personalize subsequent responses and create increasingly richer profiles about the user (Zhong et al. 2024). These learned factors may therefore influence trust calibration, as they shape the content of the answers that users receive, which may be deeply personalized and as such bring both benefits and risks (Kirk et al. 2023). Importantly, the dynamics behind propositions 2 and 4 may furthermore create algorithmic feedback loops, driven by human and algorithmic behavior (Wagner et al. 2021), with both the individual and the system adjusting their behavior to each other throughout interactions, with consequences to individual attitudes and subsequent behavior. This leads to

**P4.** Agent learned factors shape trust calibration via their influence on how agents answer questions by the user, with these factors being subsequently influenced by user behavior and feedback during interactions.

These learned factors may have different effects depending on the use context. For example, emerging studies show individuals increasing self-disclosure to agents across time partly due to the longer-term memory of these agents (Jo et al. 2024), which may suggest that these agent capabilities may influence how individuals may use these agents for *emotional support*, with their memory about the user potentially generating feelings of familiarity (Volpato et al. 2026). For *information seeking*, these learned factors may increase the relevance of responses for the specific user via personalization, or suggestions of follow-up questions related to the user’s profile, and as such influence individual perceptions about the agent and ultimately trust.

### Situational factors

Individuals interact with genAI agents for a wide variety of purposes (e.g., Skjuve et al. 2024; Moravec et al. 2024; Chatterji et al. 2025). Each context of use may imply a different type of intended use (Cohen et al 1998; Hoff and Bashir 2015), and we argue different levels of risk or vulnerabilities—i.e., using a genAI agent to tell a joke may be less risky and follow different expectations for trust calibration compared to asking it for health-related advice. Relatedly, different use contexts may also differ in terms of which trustworthiness dimensions (e.g., safety, epistemic authority, fairness, privacy, and transparency) may be relevant, or even be operationalized (e.g., safety in health-related use may be operationalized differently than safety in entertainment-related use). In addition, user perceptions and behavior as well as system behavior may be different depending on the context. As such, we propose

**P5.** Situational factors shape trust calibration as different contexts influence the relevance of different risks and trustworthiness, also conditioning individual usage and perceptions, and system behavior.

Situational factors are, by definition, context-specific, and as such should be carefully considered per use context. A case in point is what may be considered a risk and what dimensions of trustworthiness are relevant. For *information seeking*, on the one hand, one can argue that among the risks (Weidinger et al. 2022) posed by the models that power these agents, misinformation harms may be particularly relevant, and dimensions including truthfulness, epistemic authority, and accuracy may be the most relevant for information seeking, and as such guide the assessment of their trustworthiness. For *emotional support*, on the other hand, user nudging or manipulation may be a prominent risk, as well as compromising privacy, and as such fairness and privacy may be relevant trustworthiness dimensions. Moreover, some trustworthiness dimensions may take different forms depending on the use context: safety in the case of *emotional support* may be related to how the advice provided by the agent may be applicable and safe for the specific user (Wu et al. 2026), whereas for *information seeking*, it may have to do with the accuracy and safety of the information provided.

### Broader environmental factors

Trust calibration takes place along a broader societal-institutional context, which influences both how the organizations that develop and deploy these agents operate, as well as individual perceptions and expectations about these agents. For example, genAI agents may be subject to different legislation or normative frameworks across jurisdictions (Poncibò and Ebers 2025) which may influence how organizations operate, and potentially individual expectations about technology given their trust in regulators (as suggested by the link between institutional trust and trust in AI, as seen in e.g., Araujo et al. 2023). Broader societal debates as well as the media discourse may also shape what individuals expect from, interact with and perceive technological artifacts (Fisher and Wright 2001; Lane et al. 2023; Nguyen and Hekman 2024; Wald et al. 2026). Moreover, the broader institutional context, including broader institutional claims made by organizations when it comes to the technology (e.g., as seen in the logic of AI safety securitization; Spelda and Stritecky 2025), should be taken into account. As such, we propose

**P6.** Broader environmental factors shape trust calibration in genAI agents by influencing both how individuals perceive and use these agents, as well as how the organizations behind these agents operate.

Unlike the previous factors, these environmental factors may be less context-specific as they act as a background set of characteristics that influence how individuals generally approach the technology within the broader context. However, if the broader societal or institutional discourse—for example, the news coverage about these agents, or the marketing discourse of the organizations behind these agents—privileges certain frames or aspects of use (e.g., portraying these agents as “intelligent assistants”, “friendly companions”, or solutions to certain societal issues), then one could arguably expect that such discourse may have different influence in trust calibration dynamics depending on the context.

### The trust calibration process

Finally, in line with earlier scholarship (e.g., Lee and See 2004; Hoff and Bashir 2015; Wischnewski et al. 2023), trust calibration is positioned as a process in which individuals are adjusting their trust (or perceived trustworthiness) to match the system behavior (i.e., its actual trustworthiness). This is influenced by the factors proposed throughout the integrative model, with actual trustworthiness being conditioned by situational factors—i.e., the context of use, determining which trustworthiness dimensions are relevant, and how. We propose

**P7.** Trust calibration in genAI agents is a process in which users adjust their levels of trust in the agent (perceived trustworthiness) depending on the trustworthiness of its behavior, influenced by dispositional, learned, and situational factors.

## Discussion

Our knowledge about trust calibration in technology largely comes from research that has primarily focused on the user acting as an *operator* actively being asked to evaluate the system *output* for a specific task, in contexts such as security and safety, transportation, military, production, gaming, or medicine (for an overview, see Wischnewski et al. 2023). The emergence of genAI agents and their increasing use by individuals in their daily lives across a wide variety of contexts, however, requires an integrative framework to understand how individuals develop and calibrate trust in this technology.

In the current paper, we proposed a first step towards such an integrative framework, based our current body of knowledge on trust in technology; yet, this framework should be further refined, validated, and extended by empirical research that addresses the key challenges outlined above. Specifically, future research should adopt a user-centric view, investigating the use of genAI agents by individuals in their daily lives in its full complexity, integrating different use purposes and investigating the impact of individual differences as well as individual use trajectories (challenge 1). In addition, empirical research should build on the existing body of knowledge on trust calibration research (Wischnewski et al. 2023) and develop methods to assess the system’s actual trustworthiness that matches the fine-grained level of individual use and the dynamic nature of the system behavior, and adopt context-specific dimensions of trustworthiness (challenge 2). When considering trust in genAI agents, research should not only specify who is the trustee being investigated (e.g., is it the genAI agent, the technology, the answers provided?) but also acknowledge that the complex network of actors behind the technology may also influence individual trust calibration in different ways (challenge 3). In this, research should consider the complex ontological nature of these agents, therefore continue investigating the different ways in which individuals may conceptualize and relate to these agents as partners, distinct entities, or tools (challenge 4). Finally, research should adopt designs that can account for the over-time, dynamic and non-linear nature of trust calibration (challenge 5). By building upon and refining this integrative model towards trust calibration and, importantly, testing it empirically, future research may be able to extend our body of knowledge on trust in technology to empower future interventions to address the risks of both technology misuse and disuse of genAI agents by individuals in their daily lives.

## Data Availability

No datasets were generated or analysed during the current study.

## Footnotes

1 While a synthesis of possible dimensions of (actual) trustworthiness from generative AI is beyond the scope of this paper, guidelines for trustworthy AI have been suggested including the EU’s Ethics Guidelines for Trustworthy AI (AI High Level Expert Group 2019) including human agency and oversight, (technical) robustness and safety, privacy and data governance, transparency (and explainability), diversity, non-discrimination and fairness, societal and environmental wellbeing, and accountability, with other taxonomies focusing on a subset of these concepts (e.g., Toreini et al. 2019; Kaur et al. 2022). Emerging research on trustworthy LLMs (e.g., Liu et al. 2022; Huang et al. 2024) suggests similar dimensions such as safety and robustness, non-discrimination and fairness, privacy, accountability, transparency and explainability, as well as additional dimensions such as truthfulness and accuracy or (machine) ethics (Huang et al. 2024), and environmental well-being (Liu et al. 2022). Crucial to the current discussion is the acknowledgement that these dimensions may take different shapes and levels of importance depending on the use context, as we outline in Sect. 4.

2 We sincerely thank an anonymous reviewer for raising this point.
